# *CCD* ^2^: design constructs for protein expression, the easy way

**DOI:** 10.1107/S2059798321005891

**Published:** 2021-07-29

**Authors:** Andrea Giovanni Murachelli, George Damaskos, Anastassis Perrakis

**Affiliations:** a Oncode Institute and Department of Biochemistry, Netherlands Cancer Institute, Plesmanlaan 121, 1066 CX Amsterdam, The Netherlands; b Department of Biochemistry, Netherlands Cancer Institute, Plesmanlaan 121, 1066 CX Amsterdam, The Netherlands

**Keywords:** protein analysis, meta server, *CCD* ^2^, protein expression, expression construct design

## Abstract

Studying the function or structure of proteins usually requires the generation of many protein-truncation constructs for recombinant expression, which is a tedious and error-prone job. *CCD* ^2^ is a software tool designed to facilitate and automate this task. *CCD* ^2^ helps scientists by aggregating the information necessary to design protein-expression constructs. This information includes sequence conservation, secondary structure prediction, domain(s) and disorder detection, post-translational modifications and information on similar (domain) structures that are available in the Protein Data Bank. *CCD* ^2^ then allows users to easily choose the boundaries for protein constructs and automatically generates the primers necessary for construct amplification by polymerase chain reaction. Finally, *CCD* ^2^ provides a quick analysis of the properties of the chosen constructs, together with their DNA vector maps for bookkeeping. The features of *CCD* ^2^ are discussed step by step, showing that it can be a useful tool for laboratories that engage in recombinant protein production for any type of experiment, and in particular for structural biology studies.

## Abstract 2

*CCD* ^2^ is a software tool that aggregates sequence information for protein sequences (conservation, structure prediction, domain and disorder detection), enabling informed choices for expression-construct design, the single-click generation of PCR primers for cloning and easy data tracking.

## 1. Introduction   

Proteins, especially from eukaryotes, are modular machines comprising multiple domains, often connected by flexible regions. Most structural biology projects require the generation of multiple truncation constructs in order to explore recombinant expression, solubility, crystallizability or the functional properties of a target protein or macromolecular complex. Generation of a protein construct has two phases. Firstly, the constructs must be designed based on features of the sequence of the protein of interest. The aim here is to find suitable cutting points that are most likely to preserve protein folding and solubility while retaining the desired functional properties. Secondly, once the truncation constructs are known, amplification primers must be designed to amplify the relevant DNA sequence, which will then be cloned into a suitable recombinant expression vector. Although all of the information necessary for protein-construct design is available online, aggregating it and mapping it onto the protein of interest is rather tedious. Furthermore, truncation points are decided on the protein sequence, but primer design requires working with the DNA sequence. Mapping protein residues to the DNA sequence and designing primers with suitable chemical properties and appropriate cloning adaptors is trivial, but is error-prone and time-consuming. *ProteinCCD* (*Crystallization Construct Designer*; Mooij *et al.*, 2009 ▸), a Java, browser-based tool that we previously designed, aggregated many sequence-analysis tools in a single interface, allowing the user to generate PCR primers automatically starting from the protein sequence. However, technological changes have rendered *ProteinCCD* inoperable in modern browsers and obsolete.

*CCD* ^2^ (*Crystallization Construct Designer* 2) is the successor to *ProteinCCD*, using modern technology, but most importantly offering a largely expanded set of functionalities, features and tools.

## 2. Methods   

### 2.1. Architecture   

*CCD* ^2^ comprises of two parts: a user-facing, graphical user interface (GUI) and a server-side backend that is responsible for data gathering and manipulation. The GUI (whose functional core is also used for *LAHMA*; https://lahma.rhpc.nki.nl; van Beusekom *et al.*, 2021 ▸) consists of an interactive web page written in JavaScript/jQuery and styled with the Bootstrap CSS/html libraries. Such an arrangement allows easy extensibility and compatibility with all modern browsers. The backend is written in Python 3.6 and uses Flask (Ronacher, 2010 ▸) to expose a RESTful API to the frontend. To improve network performance, requests to external servers are asynchronously parallelized by both the frontend (AJAX) and the backend (AIOHTTP).

*CCD* ^2^ requires several third-party tools for data analysis. The software programs *IUPred* (Dosztányi *et al.*, 2005 ▸) and *NCOILS* (Lupas *et al.*, 1991 ▸) and the multiple sequence-alignment tool *MUSCLE* (Edgar, 2004 ▸) are executed locally and can be installed using operating-system-specific package managers and repositories (for example apt-get on Ubuntu). *Predator* (Frishman & Argos, 1996 ▸) can be obtained from http://ftp.ebi.ac.uk/pub/software/unix/predator/. The *DisEMBL* disorder-prediction tool (Linding, Jensen *et al.*, 2003 ▸) has been reimplemented for Python 3.6+ using the original code distributed by the *DisEMBL* authors as a starting point. The Savitsky–Golay smoothing algorithm is copyright by SciPy Developers and is distributed under its own licence. Predictions from *GlobPlot* (Linding, Russell *et al.*, 2003 ▸), the Pfam database (El-Gebali *et al.*, 2019 ▸), *SMART * (Letunic *et al.*, 2021 ▸), *NLS Mapper* (Kosugi *et al.*, 2009 ▸), *UniProt* (The UniProt Consortium, 2019 ▸) and *Phosphosite* (Hornbeck *et al.*, 2004 ▸) are gathered through the respective web servers. Secondary-structure predictions using *HNN* (Guermeur, 1997 ▸), *DPM* (Deléage & Roux, 1987 ▸) and *MLRC* (Guermeur *et al.*, 1999 ▸) are gathered via the NPS@ server (Combet *et al.*, 2000 ▸). Similarity searches against the PDB (Berman *et al.*, 2003 ▸) and SwissProt (The UniProt Consortium, 2019 ▸) databases are performed against a local copy of these databases using NCBI *BLAST+* (Camacho *et al.*, 2009 ▸; Altschul *et al.*, 1990 ▸). A *CCD* ^2^ script (update_db.py) takes care of installing and/or updating these local database reposi­tories.

**Figure 1:**
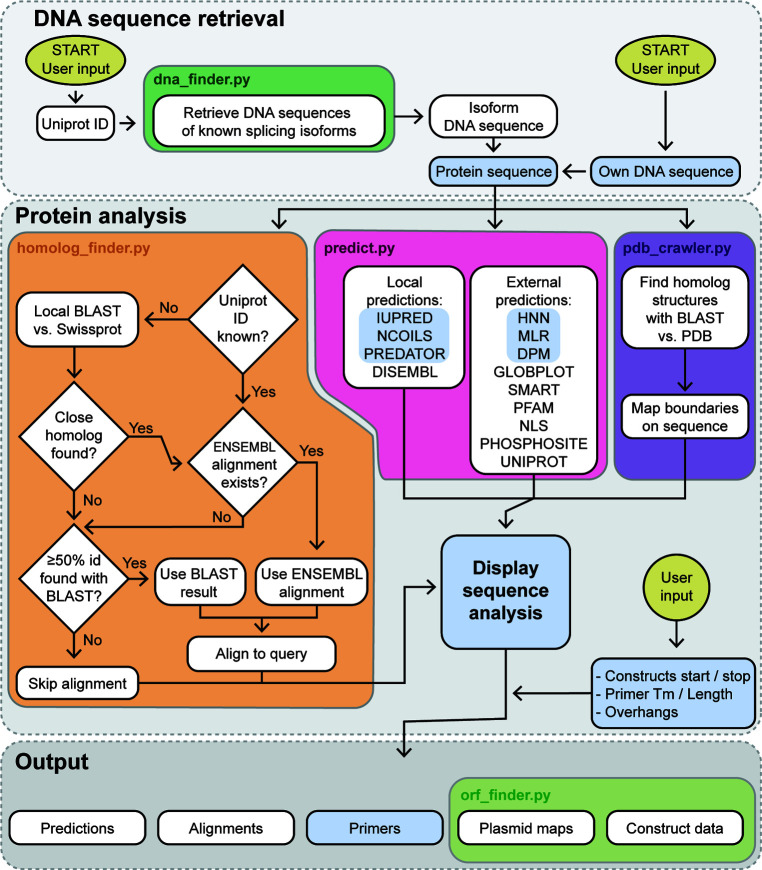
Flowchart of *CCD* ^2^. This flowchart shows the decision path/pipeline implemented by *CCD* ^2^. Points where user input is required are indicated by gold ellipses. Light-blue shaded boxes or highlighting show features that were present in the old *ProteinCCD* (Mooij *et al.*, 2009 ▸). Coloured boxes indicate which modules of the backend are responsible for each operation.

Internally, *CCD* ^2^ implements a pipeline that is summarized in Fig. 1 ▸. To improve parallelization and maintainability, different tasks are fulfilled by different modules of the backend (coloured boxes in Fig. 1 ▸), each accessible through a separate REST call.

### 2.2. Web-server implementation, installation and code availability   

*CCD* ^2^ is accessible at the URL https://ccd.rhpc.nki.nl hosted by the Netherlands Cancer Institute Research High-Performance Computing Facility.

*CCD* ^2^ can also be run locally on a Linux-based machine (tested on Ubuntu version 20.04). The source code of *CCD* ^2^ is available at https://github.com/ProteinCCD2/ProteinCCD2 and is free for noncommercial use (licencing terms are available at the code repository site). Some external tools are covered by different licencing arrangement and should be obtained by the user in accordance with the term of the respective licences.

Installation of *CCD* ^2^ is straightforward and is explained in the README.MD file provided with the distribution. Python environment consistency is maintained using Anaconda virtual environments.

## 3. Results   

In this section, we will describe the functions of *CCD* ^2^ following the natural order of user interaction schematized in Fig. 1 ▸. We will also provide some general tips about protein-construct design.

In our experience, a successful protein construct fulfils three related requirements: (i) it is recombinantly expressed (ideally at high levels), (ii) it is (highly) soluble and (iii) it is conformationally stable or at least constrained. (i) and (ii) are requirements for recombinant expression and biochemical, biophysical and many other functional assays, whereas (iii) is a requirement for a high-resolution structure by X-ray crystallography or by single-particle cryoEM. *CCD* ^2^ can help efficient recombinant expression by providing a quick and easy way to clone the user’s construct with different tags and in different hosts (see Section 3.4) and by facilitating relative bookkeeping (see Section 3.5). The solubility requirement (ii) is achieved when proteins are correctly folded and do not expose an excessive hydrophobic surface to the solvent. Due to the modular nature of proteins, this is true when a truncation cuts between, but not within, protein domains and structural elements. For structural biology and requirement (iii), one would also prune unstructured regions (*i.e.* regions that are not part of a folded domain) to limit the conformational freedom of the construct. If one is interested in intrinsically disordered proteins, and expressing disordered regions is instead the target, unstructured regions would be cloned instead. Either way, protein-construct design boils down to identifying domains and disorder in proteins. A main goal of *CCD* ^2^ is to collate and display at a glance all of the information useful for domain identification.

### 3.1. Identifying the DNA sequence of the protein of interest   

**Figure 2:**
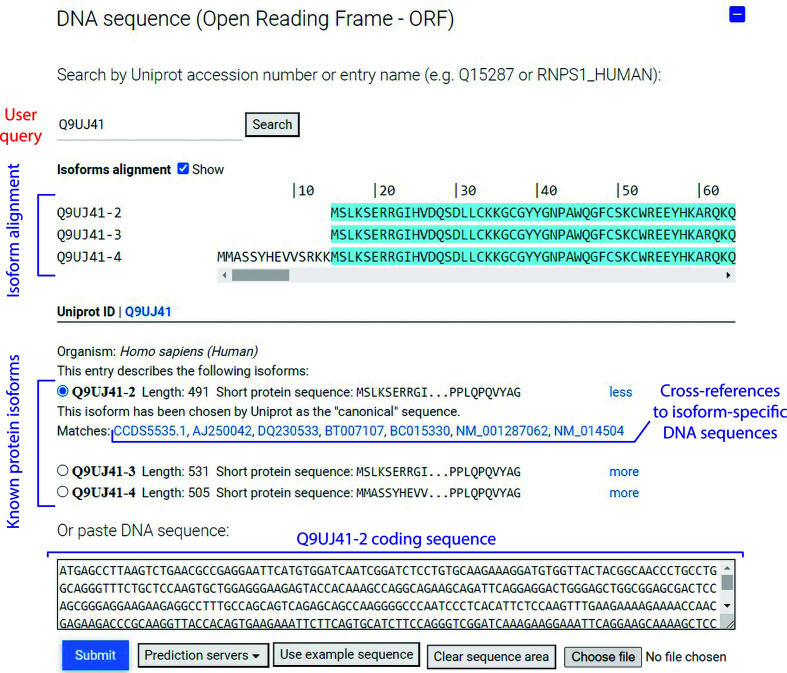
*CCD* ^2^ can automatically match UniProt protein isoforms with their encoding DNA. An annotated screenshot of the user interface of *CCD* ^2^ is depicted, showing the data retrieved for UniProt entry Q9UJ41 (human RABGEF5). The three existing isoforms of Q9UJ41 are aligned to show their differences. For each isoform, the length and matching coding DNA sequences are shown. DNA cross-references link to the entry in the respective database. Choosing an isoform (by clicking on its radio button) will automatically paste the coding DNA into the DNA sequence window, allowing *CCD* ^2^ to use it for primer design. The user can also paste their own DNA sequence, if necessary. For ease of display, some white space in this figure has been trimmed compared with the normal *CCD* ^2^ display.

The first step required for construct design is to retrieve and analyse the sequence of the protein of interest (POI). However, since the final objective is to generate cloning primers, *CCD* ^2^ needs to start by knowing the DNA sequence that codes for the POI (Fig. 2 ▸). Two options are available. The user can paste their own DNA sequence into the GUI and start the workflow from there. This is the only option available in the case where the POI is coded by an ORF that is non-natural (*i.e.* codon-optimized) or by an ORF that is not present in the UniProt database. However, if the POI is coded by a natural sequence whose translation has been deposited in the UniProt database, *CCD* ^2^ can query UniProt using a user-provided identifier (for example Q9UJ41) or mnemonic accession code (for example RABX5_HUMAN). From the UniProt entry, *CCD* ^2^ can automatically determine which isoforms of the POI are reported and match them to appropriate DNA sequences (open reading frames; ORFs) by querying the cross-referenced nucleotide databases. UniProt protein sequences are determined by consensus and curation (https://www.uniprot.org/help/canonical_nucleotide), meaning that there is no one-to-one match between DNA primary database accessions and protein isoforms. CCD^2^ simply gathers all the cross-referenced DNA sequences, translates them and matches them to isoforms at the protein level. No attempt is made to compare the raw DNA sequences for silent single-nucleotide polymorphisms, because these are rare and are extremely unlikely to affect the eventually generated primers. Imperfect protein sequence matches of up to three single amino-acid substitutions are shown to the user if no perfect match can be found for an isoform, along with a detailed notice about the sequence differences. For bacterial proteins, *CCD* ^2^ can parse multicistronic genes and genomic sequences (where the entry does not exceed 1 Mb in download size).

Once isoform matching is complete, the user is prompted to choose which isoform they wish to use for downstream analysis and primer generation. Cross-references to the primary DNA databases are provided for each isoform (Fig. 2 ▸). For easier visual reference, an alignment of the different isoforms is also provided. Inspection of the differences between isoforms can suggest viable truncation positions and hint at domains that might be swapped in or out among isoforms.

### 3.2. Creating and visualizing a report on sequence conservation   

Domains are functionally and structurally constrained, and are thus evolutionarily conserved. Disordered and linker sequences are under looser evolutionary pressure and mutate more frequently, unless they are of specific functional importance. In general, in a multiple sequence alignment, well ordered domains will appear as contiguous stretches of conserved residues, whereas linker and disordered regions will show higher divergence. *CCD* ^2^ attempts to find and display a multiple sequence alignment of the POI using three approaches. If a UniProt ID is provided by the user, and this ID can be mapped to a pre-calculated Ensembl alignment (Yates *et al.*, 2020 ▸), this alignment is retrieved. If no UniProt ID is available or provided, *CCD* ^2^ performs a *BLAST* search against a local copy of the SwissProt database and looks for up to five hits that have an identity of >95% with the POI. *CCD* ^2^ then queries Ensembl and looks for pre-calculated alignments for any of these hits. If such a hit exists, it is considered a homolog to the POI and the corresponding Ensembl alignment is retrieved. If this approach also fails, *CCD* ^2^ displays results of the *BLAST* search that (i) have an *E*-value of >0.001 and (ii) represent sequence coverage of the POI of ≥75%. Requiring a high sequence coverage is likely to find and display true orthologues of the POI (rather than showing sequences of loosely related proteins that simply share a single domain with the POI). Then, the POI isoform chosen by the user is aligned with the homolog sequences using *MUSCLE* (Edgar, 2004 ▸).

**Figure 3:**
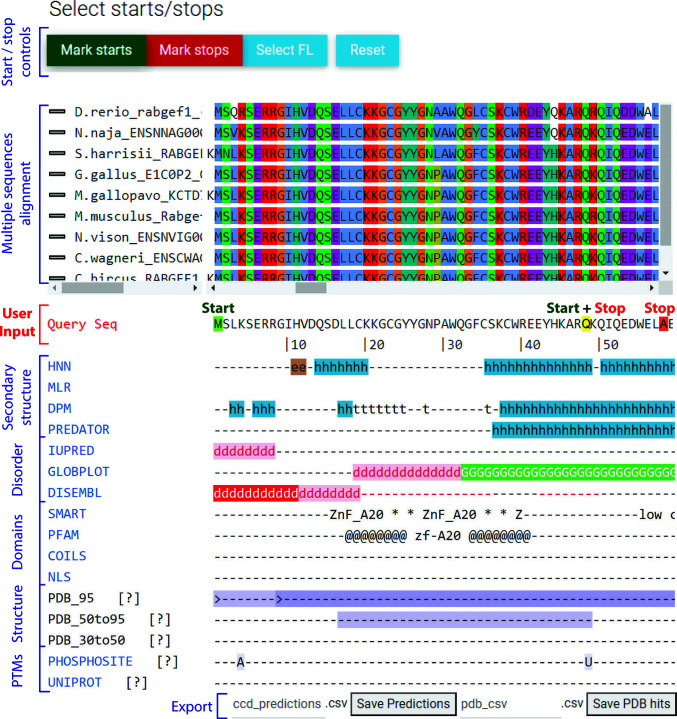
*CCD* ^2^ shows the results of many sequence analyses, facilitating the choice of construct boundaries. *CCD* ^2^ displays the query protein sequence (for example Q9UJ41 isoform 2) between a multiple sequence alignment (usually derived from Ensembl) and the results of multiple sequence analyses. The vivid colours allow an intuitive, visual interpretation of the results; the vertical alignment allows easy mapping of the analyses to the sequence. The user needs only choose where constructs should start or end by clicking on the query sequence. Green boxes indicate start points, red boxes indicate stop positions and yellow boxes indicate residues that are both a start and a stop point. Note that the truncation point at Ala58 was added for illustrative reasons and is unlikely to be a good truncation boundary, since it cuts in a long helix within a globular domain. Q9UJ41 1–48 is expressed, but does not readily crystallize (data not shown). Prediction legend: e, β-strand; h, helix; t, loop; d, disordered; G, globular; * or @, span of the predicted domain (for example ZnF_A20); >, start position of a known structure; A, acetylation; U, ubiquitination.

The Ensembl or on-the-fly constructed multiple sequence alignment is then displayed in the GUI (Fig. 3 ▸, top) and coloured by conservation using the *ClustalX* scheme (Larkin *et al.*, 2007 ▸). By default, only sequences belonging to specific pre-chosen species are displayed. These species are chosen using the following two criteria: (i) they have high-quality genome sequences and (ii) they sample all main phylo­genetic classes in order to provide a wide view of the evolutionary diversity of the POI (a full list is available at https://ccd.rhpc.nki.nl/species). The user has the option of showing the entire alignment if they wish. Furthermore, if the alignment comes from Ensembl, the user has the option of selecting which types of homologs are displayed (one-to-one, one-to-many, many-to-many homologs and paralogs, as defined by Ensembl; http://www.ensembl.org/info/genome/compara/homology_method.html).

For user convenience, whenever possible, Ensembl and UniProt identifiers are renamed to indicate their organism of origin and gene name more clearly; for example ENSMUSG00000006715 is renamed to M.musculus_gmnn_ (H3BLK4_MOUSE), indicating that this is the mouse product of the *gmnn* gene, whose UniProt accession is H3BLK4_MOUSE. Alignments can be downloaded in FASTA format for bookkeeping and/or further analysis in external tools.

### 3.3. Aggregating and visualizing sequence-information data   

All the different data are gathered from various software, either locally or using web services, collated and displayed at below the multiple sequence alignment and the POI sequence (Fig. 3 ▸, bottom). Below we discuss all the different types of information collected and displayed by *CCD* ^2^.

#### 3.3.1. Secondary-structure prediction   

Domains have a high content of secondary structure, while disordered regions do not. *CCD* ^2^ runs the sequence through four secondary-structure prediction algorithms [*HNN* (Guermeur, 1997 ▸), *DPM* (Deléage & Roux, 1987 ▸), *MLRC* (Guermeur *et al.*, 1999 ▸) and *Predator* (Frishman & Argos, 1996 ▸)]. These secondary-structure prediction methods are reasonably reliable and quick. Their results are displayed together, so that the user can derive a consensus view. Consecutive stretches of consensus secondary structure indicate domains.

#### 3.3.2. Disorder prediction   

Disordered regions often have low-complexity, repetitive sequences. Additionally, polar and charged amino acids are overrepresented in disordered regions (Dyson, 2016 ▸). *CCD* ^2^ gathers disorder and globular region information using *IUPred* (Dosztányi *et al.*, 2005 ▸), *DisEMBL* (Linding, Jensen *et al.*, 2003 ▸) and *GlobPlot* (Linding, Russell *et al.*, 2003 ▸). The SMART database (Letunic *et al.*, 2021 ▸) is also used to display low-complexity regions. Cuts in the constructs should encompass, but not cut within, predicted globular regions. Trimming terminal disordered regions is generally required for crystallization, and might lead to more homogeneous protein preparations owing to reduced proteolytic degradation.

#### 3.3.3. Domain detection   

*CCD* ^2^ highlights known domains in the protein sequence by querying the SMART (Letunic *et al.*, 2021 ▸) and Pfam (El-Gebali *et al.*, 2019 ▸) domain-fingerprint databases. Additionally, *CCD* ^2^ performs a *BLAST* search (Altschul *et al.*, 1990 ▸) against a local copy of the Protein Data Bank (PDB), reporting hits at three different levels of similarity. The prediction ‘PDB_95’ highlights the parts of the POI sequence that have an identity of ≥95% to a solved structure in the PDB, thus indicating that parts of the POI (or of a very close homologue) have been experimentally determined. The boundaries of the expression constructs deposited in the corresponding PDB structures are also indicated on the POI sequence. Hovering the cursor over the construct boundaries (marked with ‘>’ or ‘<’ for a start or stop position, respectively) will display the PDB code and chain of the matching structures.

These are experimentally validated, effectual boundaries for truncation constructs. The predictions ‘PDB50_to_95’ and ‘PDB30_to_50’ similarly highlight parts of the POI sequence that have *BLAST* hits against the PDB with identities between 95% and 50% and between 50% and 30%, respectively. These portions of the POI sequence are homologous to known structures, indicating the likely existence and approximate boundaries of a folded domain. All of the results of the search against the PDB can be downloaded for further analysis by clocking on the ‘Save PDB hits’ button. These include the PDB code, sequence coverage and percentage identity for each matching hit.

#### 3.3.4. Coiled-coil detection   

Coiled coils are very common structural domains that often mediate protein–protein interactions. *CCD* ^2^ searches for coiled coils by querying the SMART database and by direct prediction with *NCOILS* (Lupas *et al.*, 1991 ▸). Truncation within coiled coils is possible (see, for example, Ciferri *et al.*, 2008 ▸), although trial and error is necessary.

#### 3.3.5. Detection of other functional elements   

*CCD* ^2^ further detects the presence of putative nuclear localization signals (NLS) using *NLS* (Kosugi *et al.*, 2009 ▸). The presence of an NLS can influence expression in eukaryotic systems. However, NLSs are low-complexity, generally disordered sequences, so their removal can positively affect crystallization.

If experimentally validated post-translational modifications (PTMs) are recorded in the UniProt entry for the sequence of interest, these are displayed. UniProt covers a wide variety of possible PTMs, including glycosylation, disulfide bridges, cross-links (intra-chain and to other proteins such as ubiquitin), chemical modification of amino acids and more. These modifications are indicated with a single-letter code including, for example, ‘A’ for acetylation, ‘^’ for a disulfide link, ‘+’ for multiple known modifications *etc.* (a full legend can be found on the tutorial page at https://ccd.rhpc.nki.nl/tutorial). Hovering the cursor over each letter will display more precise information about each modification. Because UniProt annotations always refer to the sequence of the canonical isoform, these annotations are disabled if the user has selected an alternative splicing variant, to avoid sequence discrepancy.

When data are available (human, rat and mouse proteins), *CCD* ^2^ also queries the Phosphosite Plus database (Hornbeck *et al.*, 2004 ▸) for the presence of experimentally validated post-translational modifications (PTMs) on the sequence. Annotations follow the same notation as for UniProt above. Hovering over each annotation provides further information about the underlying data.

PTMs are added by enzymes and require physical accessibility to be attached. Thus, the presence of PTMs can hint at disordered, highly accessible linker regions or at least solvent-exposed residues (Dyson, 2016 ▸). PTMs can also inform about the functionality of truncation constructs.

### 3.4. Designing protein constructs and single-click generation of DNA primers   

With all the necessary information available, the user can choose where truncation constructs should start or stop by clicking start and stop points on the sequence of the POI (Fig. 3 ▸, middle). The clicked amino acid is always included in the final construct. Start points will generate forward PCR primers and stop points will generate reverse PCR primers. A position can be marked as being both a start and a stop.

**Figure 4:**
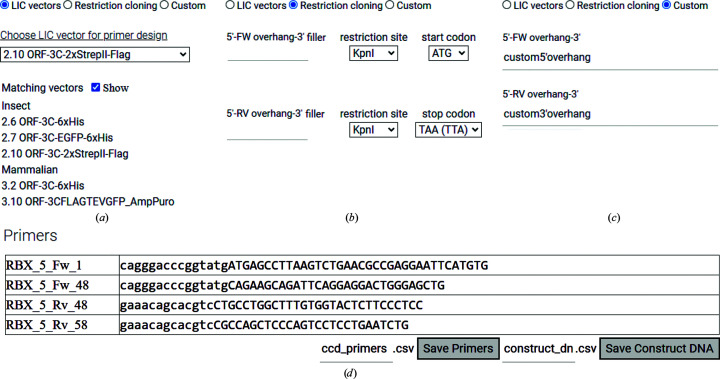
*CCD* ^2^ offers multiple choices for primer generation. (*a*) *CCD* ^2^ is (optionally) integrated with the pETNKI LIC series of vectors, which offer great versatility, since the same construct fits multiple vectors. (*b*) *CCD* ^2^ can design primers for conventional restriction cloning. (*c*) *CCD* ^2^ allows the choice of any custom overhang for primers. (*d*) *CCD* ^2^ automatically generates primers based on user-chosen boundaries on the protein sequence, melting temperature and primer overhangs. Shown here are the primers for start positions 1 and 48 and stop positions 48 and 58 (from Fig. 3 ▸), with a *T*_m_ of 65°C and overhangs for pETNKI LIC 1.1. The overhang portion of the primer is shown in lower case and the annealing portion is shown in upper case. The primers are named prefix_Fw/Rv_position, where the prefix is chosen by the user (for example RBX5), Fw stands for forward, Rv stands for reverse and ‘position’ is the chosen start/stop position. Primers can be copied and pasted into a spreadsheet or saved in comma-separated value (csv) format. The DNA sequences of the constructs resulting from all possible combinations of primers can also be saved in csv format.

PCR amplificates typically need adapter sequences to be cloned into recipient vectors. These sequences are added to the primers as ‘overhangs’ that extend beyond the primer sequence that anneals to the template DNA. *CCD* ^2^ allows the user to choose overhangs in three ways (Figs. 4 ▸ *a*–4 ▸ *c*). Firstly, the version of *CCD* ^2^ hosted on our servers is designed to work in tandem with the pETNKI series (Luna-Vargas *et al.*, 2011 ▸) of ligation-independent cloning (LIC; Aslanidis & de Jong, 1990 ▸) vectors. These vectors are suitable for mammalian, insect-cell or *Escherichia coli* expression and are designed for maximum intercompatibility, so that the same PCR amplificate can be cloned in multiple targets. *CCD* ^2^ can automatically generate PCR primers with the correct overhangs for any pETNKI vector chosen (Fig. 4 ▸ *a*). Some pETNKI vectors can be freely obtained from Addgene (https://www.addgene.org/, catalogue Nos. 108703–108710); others, which are encumbered by third-party patents, can be sourced from the Netherlands Cancer Institute protein-production facility with a material transfer agreement. When running a local copy of *CCD* ^2^, user-defined vectors can be integrated instead of the petNKI series (not shown). Alternatively, *CCD* ^2^ contains a utility to generate primer overhangs for conventional restriction cloning (Fig. 4 ▸ *b*). Finally, *CCD* ^2^ can accept user-provided custom overhangs, which may contain nonstandard sequences (*i.e.* other than the standard DNA bases ATCG; Fig. 4 ▸ *c*). In all cases, the user is notified of the final overhang sequence and of the presence of start/stop codons in the overhang (not shown). The user can also choose the properties of the primer by choosing a desired melting temperature (*T*_m_; default 65°C) or primer length. Overhangs are not considered in determining the *T*_m_. Finally, the user can also choose a name for the primers.

Using these data, *CCD* ^2^ automatically maps the user-chosen start and stop positions from the protein to the DNA sequence and generates a table with all of the primers that can be saved in spreadsheet-compatible format for bookkeeping or copied and pasted for quick ordering of the primers (Fig. 4 ▸ *d*). The amplified DNA sequences resulting from all possible combinations of starts and stops (*i.e.* resulting from a start and stop primer that amplifies any portion of the protein sequence) can also be downloaded in spreadsheet-compatible format by clicking on the ‘Save Construct DNA’ button.

### 3.5. Enabling data tracking and bookkeeping   

**Figure 5:**
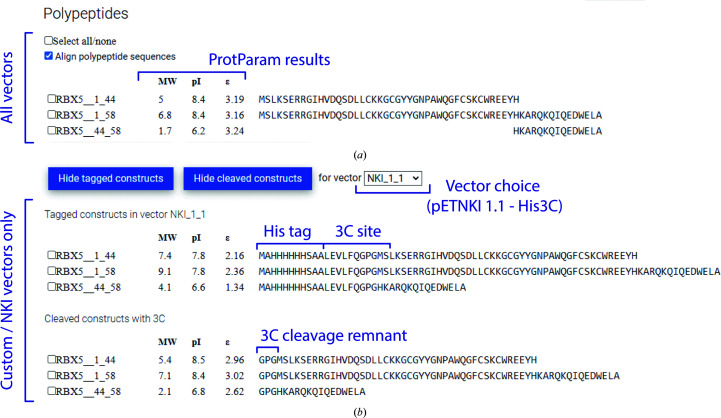
Primer generation and protein-construct analysis. (*a*) The possible constructs resulting from the primers chosen in Fig. 3 ▸ and displayed in Fig. 4 ▸(*e*) are shown here, together with their predicted molecular wight (MW), isoelectric point (pI) and extinction coefficient ɛ at 280 nm. (*b*) For pETNKI vectors (for example pETNKI 1.1), the tagged and protease-cleaved constructs are also shown, together with their predicted physical properties.

*CCD* ^2^ displays the sequence of the protein truncations that are generated by all possible start and stop combinations in a different panel, along with basic information about their predicted molecular weight (MW), isoelectric point (pI) and predicted extinction coefficient at 280 nm (Fig. 5 ▸ *a*). These are calculated with the same algorithm as used by *ProtParam* in the Expasy portal (Gasteiger *et al.*, 2005 ▸). If pETNKI vectors are chosen as cloning targets (or custom vectors are integrated in a local copy of *CCD* ^2^), *CCD* ^2^ also has the information about the sequence of each construct prior to (Fig. 5 ▸ *b*) and after (Fig. 5 ▸ *c*) proteolytic tag cleavage, and can provide further provide the sequence, molecular weight, predicted isoelectric point (pI) and expected 280 nm extinction coefficient for all generated constructs, either with attached tag or after protease digestion. All of these data can be saved in spreadsheet-compatible format for bookkeeping and to assist in protein expression and purification.

Finally, for pETNKI and custom vectors, *CCD* ^2^ can generate and save annotated plasmid maps of the chosen truncation constructs (GenBank format; https://www.ncbi.nlm.nih.gov/genbank/samplerecord/). These can be opened in any standard DNA-manipulation software and are useful as a reference to check the success of cloning.

## 4. Conclusions   

The design and cloning of constructs are frequent and time-consuming tasks in any structural biology project, and often in general biochemistry and biophysics. *CCD* ^2^ streamlines these tasks: it helps in the design of constructs by consolidating multiple informative analyses of the sequence in a single place, and it enables the user to make quick decisions about where protein truncations should be placed. Then, once the boundaries have been chosen, *CCD* ^2^ takes care of the nitty-gritty details of primer design and plasmid mapping, also providing a brief recombinant construct analysis. Overall, *CCD* ^2^ allows the user to save valuable time and reduce costly mistakes in any structural biology project.
